# Development and Optimization of *Nigella sativa* Nanoemulsion Loaded with Pioglitazone for Hypoglycemic Effect

**DOI:** 10.3390/polym14153021

**Published:** 2022-07-26

**Authors:** Tamer M. Shehata, Mervt M. Almostafa, Heba S. Elsewedy

**Affiliations:** 1 Al Bilad Bank Scholarly Chair for Food and Security in Saudi Arabia, The Deanship of Scientific Research, The Vice Presidency for Graduate Studies and Scientific Research, King Faisal University, Alhofuf 36362, Al-Ahsa, Saudi Arabia; malmostafa@kfu.edu.sa (M.M.A.); helsewedy@kfu.edu.sa (H.S.E.); 2 Department of Pharmaceutical Sciences, College of Clinical Pharmacy, King Faisal University, Alhofuf 31982, Al-Ahsa, Saudi Arabia; 3 Department of Pharmaceutics, College of Pharmacy, Zagazig University, Zagazig 44519, Egypt; 4 Department of Chemistry, College of Science, King Faisal University, Alhofuf 31982, Al-Ahsa, Saudi Arabia

**Keywords:** diabetes, nanoemulsion, *Nigella sativa*, pioglitazone, optimization, hypoglycemia

## Abstract

Diabetes mellitus (DM) is a metabolic disorder associated with an increased blood glucose level. The world health burden of DM has increased as a result of numerous causes that necessitates suitable treatment. Pioglitazone (PGZ) is a generally prescribed medication for managing type II diabetes. However, its low solubility creates complications for its formulation. Therefore, the aim of the current study was to incorporate PGZ into a nanoemulsion (NE) formulation prepared with *Nigella sativa* oil (NSO) to boost the action of PGZ. To our knowledge, no previous study has addressed the combination and synergistic effect of PGZ and NSO as a hypoglycemic NE formulation intended for oral administration. An experiment was designed to test several PGZ-loaded NE formulations, varying factors such as NSO, surfactant and co-surfactant concentrations. These factors were investigated for their influence on responses including particle size and *in vitro* release. An optimized PGZ-loaded NE was selected and examined for its morphology, kinetic activity and stability. Further, the anti-diabetic effect of the optimized formulation was evaluated using diabetically induced rats. The optimized formula exhibited a good particle size of 167.1 nm and *in vitro* release of 89.5%. A kinetic study revealed that the drug release followed the Korsmeyer–Peppas mechanism. Additionally, the PGZ-loaded NE formulation was found to be stable, showing non-significant variation in the evaluated parameters when stored at 4 and 25 °C for a period of 3 months. In vivo investigation of the PGZ-loaded NE formulation showed a significant reduction in blood glucose level, which appeared to be enhanced by the presence of NSO. In conclusion, NS-NE could be a promising nanocarrier for enhancing the hypoglycemic effect of PGZ.

## 1. Introduction

Diabetes mellitus is a public health issue and is a metabolic disorder that is a leading cause of death [1]. It is associated with variation in the homeostasis of blood glucose as a result of imperfections in insulin secretion that can lead to hyperglycemia [2]. The prevalence of diabetes is influenced by factors such as an unhealthy diet, obesity, low exercise levels, stress and genetic factors [3]. Diabetes is distinguished into two types, types I and II, with most diabetic patients suffering from type II diabetes. The complications associated with diabetes mellitus include eye problems, nerve damage, and renal and cardiovascular disease [4]. Consequently, managing high blood glucose level is crucial.

Pioglitazone hydrochloride (PGZ) is an anti-diabetic drug belonging to the thiazolidinedione group which has an insulin-sensitizing action [5]. Its capacity for the improved control of blood glucose level and lipid profile has been established, along with demonstration of tolerable side-effects [6]. However, according to its categorization in the Biopharmaceutical Classification System (BCS), it is characterized as a class II drug, which exhibits poor solubility and high permeability [7]. This presents a challenge for PGZ formulation as it necessitates the development of a dosage form that can incorporate a drug with low solubility in water.

Recently, nanotechnology has shown promise as an advanced technique for enabling the delivery of poorly water-soluble drugs [8]. Nanocarrier-based drug delivery systems are colloidal carriers for drugs with dimensions in the nano range which have been used to avoid a wide range of problems associated with conventional systems, such as poor specificity, increased toxicity and development of drug resistance [9]. Various nanocarriers have been developed and their effectiveness in encapsulating poorly water-soluble drugs has been demonstrated. Lipid-based nanocarriers represent an example of these drug delivery systems that have proved to be efficient, particularly for oral delivery [10]. Nanolipid formulations include nanoemulsions (NEs), which are formed from an oil and aqueous phase dispersed with each other in the presence of a surfactant and co-surfactant [11]. NEs have various advantages since they are safe, non-toxic, and can solubilize hydrophobic drugs and improve the physical stability of the formulation. Moreover, the nano scale of the particles can improve drug absorption and, consequently, drug bioavailability [12]. To achieve enhanced effects, NEs could be synthesized using particular drug and natural oil combinations to create a synergistic effect when given together as one formulation [13]. Moreover, there is scope for the use of natural products in treatment protocols which can promote human health due to their established safety and pharmacological behavior [14].

*Nigella sativa* (NS), renowned as black cumin, belongs to the family Ranunculaceae, and is regarded as a medicinal plant that is widely used to treat various disorders [15]. It has been demonstrated to have anti-inflammatory, analgesic, antibacterial, antioxidant, anticancer and anti-diabetic effects [16,17,18]. The evidenced therapeutic properties reflect its valuable constituents [19]. *Nigella sativa* contains various nutrients, such as protein, carbohydrate, fats and fibers, in addition to different amino acids, and minerals, including calcium, copper, iron, zinc and folic acid [20]. Moreover, NS contains up to 34% fixed oils and 2.5% essential oils. The essential oils contain numerous compounds, the most active component reported to be responsible for most NS therapeutic effects being thymoquinone [21,22]. There have been many investigations that have reported the effects of thymoquinone and NS on hypoglycemia [23]. NS influences diabetes mellitus via different pathways and mechanisms, with improved blood glucose levels probably resulting from adjustment in the production of insulin [17].

To obtain a formulation with suitable quality attributes, a quality by design (QbD) process should be employed for product manufacturing [24]. A Box–Behnken design (BBD) is an experimental model that can be used to achieve the QbD objective and assist in selecting an optimized formulation based on specific factors and their effects [25]. This study involves the application of a nanotechnology approach to develop an NE as a potential nanolipid carrier containing an anti-diabetic agent to be administered as an oral liquid preparation for patients suffering from difficulty in swallowing. Various NE formulations incorporating PGZ, an anti-diabetic agent, prepared with NSO, were fabricated using different concentrations of oil, surfactant and co-surfactant based on BBD modeling. An optimized formula was selected and evaluated for various characteristics and investigated for its hypoglycemic effect.

## 2. Materials and Methods

### 2.1. Materials

Pioglitazone hydrochloride was obtained from Carbosynth Limited, Compton, Berkshire, UK. *Nigella Sativa* oil was obtained from Oud Milano Co., Ltd. (Milan, Italy). Tween 80 was obtained from Alpha Chemika (Mumbai, India). Propylene glycol and Streptozotocin were procured from Sigma-Aldrich Co. (St Louis, MO, USA). Distearoyl phosphatidyl ethanolamine-N-[methoxy poly (ethylene glycol)-2000] (PEG-DSPE) was obtained from Lipoid LLC, (Newark, NJ, USA). All other chemicals were of analytical grade.

### 2.2. QbD Using Box–Behnken Experimental Design

A BBD was applied using the Design-Expert version 12.0 software (Stat-Ease, Minneapolis, MN, USA) in which a matrix of 15 runs was constructed and developed. The design was implemented using three factors that were investigated at three levels (−1, 0, 1), producing a (3^3^) factorial design as illustrated in Table 1. The investigated factors influencing the formulation were labelled A, B and C with respect to NSO concentration, Tween 80 concentration and PG concentration, respectively. These independent variables were examined for their effect on selected responses viewed as the dependent variables. The responses evaluated were particle size R_1_ and *in vitro* release R_2_. The data were analyzed statistically using analysis of variance (ANOVA) performed by the Design-Expert software. Further verification for the results was undertaken by generating specific modeling plots in addition to polynomial mathematical equations indicating the relation between the observed response and the main independent variables.

### 2.3. Preparation of PGZ-Loaded NE

Based on the method previously described by Khalil et al., different NE formulations were prepared based on the specified amounts of ingredients determined by the BBD; these are presented in Table 2 [26]. Two phases were prepared: an oily phase and an aqueous phase. For the oily phase 30 mg of PGZ was mixed with 50 mg PEG-DSPE, a specified amount of NSO and PG as a co-surfactant, using a classic advanced vortex mixer (VELP Scintifica, Italy). An aqueous phase of up to 10 mL containing a specified amount of Tween 80 was also prepared. The two phases were mixed with homogenization for 10 min at 20,000 rpm using a high shear homogenizer (T 25 digital Ultra-Turrax, IKA, Staufen, Germany). A PGZ-loaded emulsion was formed and subjected to 30 s sonication with a probe sonicator (XL-2000, Qsonica, Newtown, CT, USA) to obtain a homogenous NE.

### 2.4. Particle Size Measurement

The formulated PGZ-loaded NEs were evaluated for their particle size at 25 °C using a Zetasizer Nano apparatus, ZS90 (Malvern Instruments Ltd., Worcestershire, UK). The measurements were carried out carefully by assessing the dynamic light scattering [27].

### 2.5. Drug Release Study

The percentage of PGZ released from the developed NE preparation was assessed using an ERWEKA dissolution system (ERWEKA, GmbH, Heusenstamm, Germany). An amount of 1 mL of NE formulation was added to a glass tube closed from one side with a cellophane membrane (MWCO 2000–15,000) and attached to the apparatus from the other side using a paddle. The tube containing the NE sample was immersed into 500 mL of the release media, kept at 37 °C and rotated at 50 rpm. The release media consisted of 500 mL 0.1 N HCl (pH 1.2) and was applied for 2 h. Samples of 3 mL were withdrawn at specified times to detect the percentage of PGZ released and were substituted with the same volume of fresh media. The analysis was performed spectrophotometrically at λ_max_ 269 nm using a UV spectrophotometer (JENWAY 6305, Bibby Scientific Ltd., Staffs, UK) [28]. The experiment was performed in triplicate per formulation (n = 3).

### 2.6. Kinetic Study

A study was performed to predict the kinetic mechanism of drug release from the developed formulation. Briefly, the data obtained from the PGZ-loaded NE *in vitro* release study was plotted according to various kinetic models, specifically, zero-order, first-order, Higuchi, and Korsmeyer–Peppas. The most suitable model, showing the best fit of the data based on the greatest R2 value obtained, was selected [29].

### 2.7. Drug Content

A quantity of 100 µL of optimized PGZ -loaded NE formulation was diluted in 25 mL methanol and vortexed for 3 min using a vortex mixer (VELP Scintifica, Usmate Velate, Italy). The drug content was evaluated spectrophotometrically using a UV spectrophotometer (JENWAY 6305, Bibby Scientific Ltd., Staffs, UK) at λ_max_ 269 nm [7]. An identical technique was used for the blank NE (sample without drug). The drug content was then calculated using the following equation:

Drug content = (Actual/Theoretical) × 100

### 2.8. Scanning Electron Microscopy

To examine the morphological properties of the optimized PGZ-loaded NE formulation, a scanning electron microscope (SEM), (JSM-6390LA, JEOL, Tokyo, Japan) was utilized. Briefly, one drop of the NE sample was diluted and then kept on metal stubs and capped with gold. The morphology was established at different magnifications and finally examined at 10 kv [30].

### 2.9. Stability Study

The optimized PGZ-loaded NE preparation was kept in a well-closed container to be examined for its stability upon storage under different conditions. The study was performed consistent with International Conference on Harmonization (ICH) guidelines, following keeping of the formulation at refrigerator temperature (4 ± 3 °C) and ambient room temperature (25 ± 2 °C) [25].

### 2.10. Handling of Animals

A total of 18 male Wistar rats were purchased form the Experimental Animal Research Centre at King Saud University, Riyadh, KSA with an average weight of 200 ± 25 g. The rats were kept under standard conditions with an adjusted 12 h light/dark cycle at ambient room temperature. The rats were allowed free access to water and diet and were checked for blood glucose levels before proceeding with the study.

### 2.11. Ethical Approval

The in vivo experiment in this study was carried out in conformity with the Guidelines for the Ethical Conduct for Use of Animals in Research. The animal protocol of the investigation was considered and approved by the Animal Research Ethics Committee at King Faisal University, approval number (KFU-REC-2021-MAR-EA000531).

### 2.12. In Vivo Hypoglycemic Activity

#### 2.12.1. Induction of Diabetes

Animals in the current study were induced to be diabetic using Streptozotocin (60 mg/kg) via intraperitoneal injection. Streptozotocin has the capability to induce hyperglycemia within 3 days following injection since it destroys beta cells [31]. The animals were tested for their blood glucose level after fasting using a digital One Touch^®^ Ultra 2^®^ glucometer. Once the blood glucose level reached 300 mg/dL, the animals were considered diabetic and were prepared for the experimental study.

#### 2.12.2. Study Design

Three days following induction of hyperglycemia, diabetic rats were divided into four groups, each having six animals. The animals received the following treatment orally using oral gavage:

Group I: negative control group received saline orally (non-treated).
Group I: positive control group received marketed PGZ product (ACTOS^®^) (equivalent to 30 mg/kg).
Group III: rats treated with NE free from PGZ (blank NE).
Group IV: rats treated with optimized PGZ-loaded NE (equivalent to 30 mg/kg) [28].

Starting from time 0 up to 24 h, blood samples were taken from the animals’ tails and were applied to a glucometer strip to be analyzed for blood glucose level using a digital One Touch^®^ Ultra 2^®^ glucometer. The animals were fasted during 12 h of the study and fed later [32].

### 2.13. Statistical Analysis

The results were displayed as the mean ± standard deviation (SD) for at least three independent repeats. The examined groups were regarded as significantly different from each other when *p* < 0.05. All statistical analyses were confirmed using SPSS statistics software, version 9 (IBM Corporation, Armonk, NY, USA). One-way analysis of variance (ANOVA) was performed using Design-Expert version 12.0 software (Stat-Ease, Minneapolis, MN, USA).

## 3. Result and Discussion

### 3.1. Validation of BBD Data

BBD modeling resulted in 15 different PGZ-loaded NE formulations based on different levels of the selected independent variables, as exhibited in Table 2. Statistical analysis for the data was performed as shown in Table 3, revealing certain important parameters that were related to the observed response. It was apparent that the quadratic model for both responses provided the best fit model compared with other models. The *p*-value was less than 0.05 for most of the model terms for both response R_1_ and R_2_, indicating that these model terms were significant. Additionally, it was observed that the model F-values were 153.39 and 58.36 for R_1_ and R_2_, respectively, indicating that the model was also significant. With respect to the lack-of-fit F-value, it is important that this is non-significant relative to the pure error for the model to be fitted [33]. According to the data presented in Table 3, the lack of fit values were 2.78 and 1.24, with corresponding *p*-values 0.2759 and 0.4763 for R_1_ and R_2_, respectively, indicating non-significant values.

### 3.2. Analysis of Response

#### 3.2.1. Response 1: Influence of the Independent Variables on R_1_

The particle size of the prepared PGZ-loaded NE formulations was evaluated and the results are displayed in Table 2. It was observed that the particle size was influenced by the different independent variables A, B and C. The particle sizes of all the NE formulations ranged from 166 ± 3.6 up to 319 ± 5.4 nm. It was observed that increasing concentration of NSO from 1 g to 2 g was associated with a parallel increase in NE particle size, possibly due to increase in the dispersed phase. Another interpretation is that by increasing the oil concentration, disruption of the particles was reduced, and, hence, the rate of collision could increase, leading to coalescence and aggregation of droplets. This is in agreement with Zainol et al., who confirmed that increasing the concentration of oil resulted in increasing the particle size of a palm-based nanoemulsion containing Levodopa [34]. However, the particle size was influenced by the surfactant and co-surfactant concentration using the same NSO concentration. Using 1 g NSO, particle size was small when using 0.75 g of Tween 80 and 0.75 g of PG (166 nm), while it was larger (181 nm) when using 0.25 g Tween 80 and 0.75 g PG. It was concluded that higher surfactant and co-surfactant concentration led to lowering in particle size of the formulated NE. These findings can be interpreted based on lowered interfacial tension with increasing surfactant concentration and consequent decrease in the particle size of the NE [35]. The previous data was confirmed with the following mathematical equation

R_1_ = 191 + 60.5 ∗ A − 19.625 × B − 5.125 × C − 8.5 × AB − 1.5 × AC + 1.75 × BC + 23.125 × A^2^ + 19.875 × B^2^ + 19.375 × C^2^ (1)

**Figure 1:**
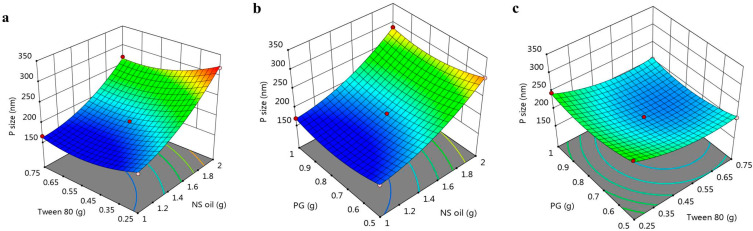
3D response surface plot representing the effect of independent variables (**a**) NSO and Tween 80, (**b**) NSO and PG, and (**c**) Tween 80 and PG on particle size (R_1_).

**Figure 2:**
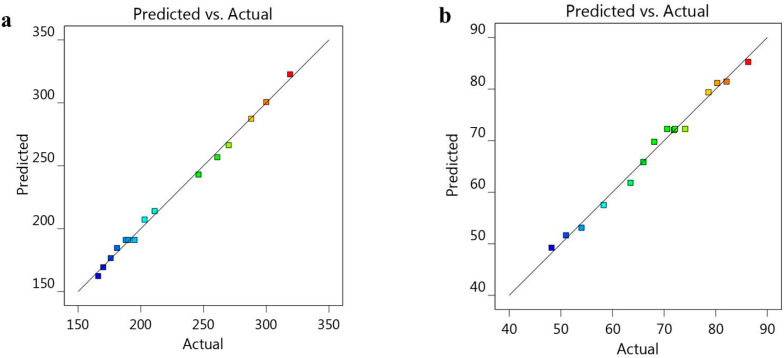
Linear correlation plot between predicted versus actual values representing the effect of the independent variables on (**a**) particle size R_1_, and (**b**) the *in vitro* release study R_2_.

**Figure 3:**
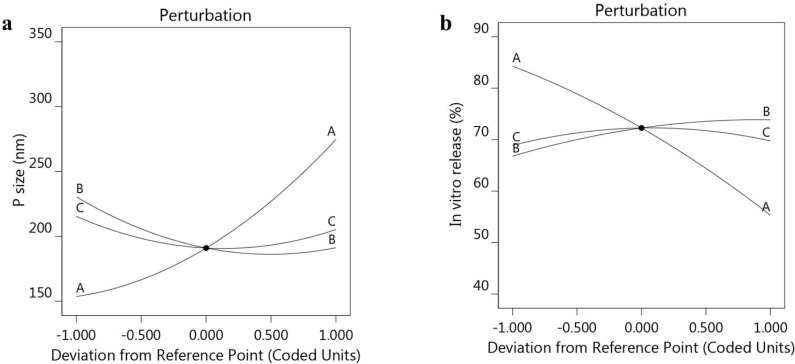
Perturbation plots displaying the influence of each independent variable alone on (**a**) particle size R_1_, and (**b**) *in vitro* release R_2_.

The positive sign of NSO (A) indicates a parallel influence; however, the negative sign for the B and C factors indicates that there was an inverse relation between these factors and the response R_1_. Further, as displayed in Figure 1a–c, a 3D response surface plot confirmed the previous relation between the three factors and the response R_1_. Additionally, Table 3 and Figure 2 illustrate a linear correlation between the predicted and actual data. This was confirmed by the value of the adjusted (0.9899) and predicted R^2^ (0.9519), with reasonable agreement between them. Moreover, perturbation plots, shown in Figure 3a, were generated to clarify the effect of each factor on the selected response if all other factors were kept constant [36]. It was apparent that factor A had a more noticeable effect on R_1_ than the other two factors, B and C, with the curvature indicating the sensitivity. Additionally, the direction of the perturbation plot confirmed that factor A had a synergistic effect on particle size R_1_, while factors B and C had antagonistic effects.

#### 3.2.2. Response 2: Influence of the Independent Variables on R_2_

An *in vitro* release study was conducted for all PGZ-loaded NE formulations with release values ranging between 48.2 ± 3.1 and 86.3 ± 2.9% after 12 h, as shown in Table 2. It was clear that NSO indirectly affected the percentage of PGZ released from the NE formulation, with increasing NSO resulting in a lower R_2_ percentage. This might be attributed to the larger particle size, since increasing oil concentration would cause a relative increase in NE particle size, providing a small surface area, lowering the percentage release from the formulation [37]. However, with a constant concentration of NSO, it was noted that increasing surfactant (B) and co-surfactant (C) concentrations enhance PGZ release from the developed NE. This may have been a result of the surfactant lowering the interfacial tension between the formulation and the surrounding aqueous media of release. This would allow for more wetting of the drug, facilitating penetration, which would increase the rate of drug released from the formulation [38]. The obtained mathematical equation emphasized the earlier noticed facts where factor A had a negative sign indicating an opposed effect, in contrast to the other two factors, B and C, that had a positive sign, indicating synergistic action.

R_2_ = 72.2667 − 14.475 × A + 3.5375 × B + 0.4375 × C + 0.6 × AB + 0.3 × AC − 1.575 × BC −2.47083 × A^2^ − 1.94583 × B^2^ − 2.94583 × C^2^ (2)

**Figure 4:**
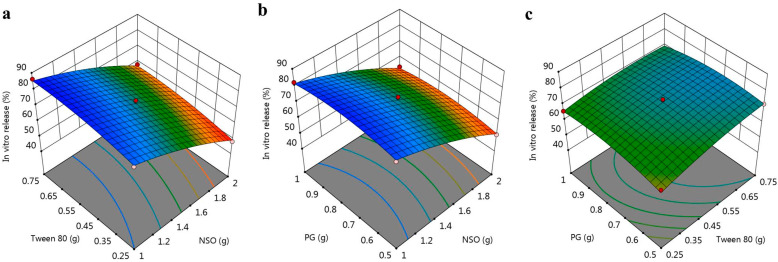
3D response surface plot representing the effect of independent variables (**a**) NSO and Tween 80, (**b**) NSO and PG, and (**c**) Tween 80 and PG on *in vitro* release (R_2_).

For further confirmation, the BBD software was used to generate certain graphs to highlight the relation between the selected independent variables and the examined *in vitro* release response R_2_. As shown in Figure 4a–c, the 3D-response surface graphs illustrate the integrated relation between the factors and response R_2_, rather than displaying individual data points [39]. Furthermore, Figure 2b shows the linear correlation between the A, B and C independent variables and the observed R_2_. Additionally, Table 3 shows the values of the adjusted and predicted R^2^ of 0.9736 and 0.8945, respectively. These values were closely allied to each other and were in reasonable agreement, as shown in Figure 2b. The perturbation graph displayed in Figure 3b, strongly supports the above findings. It was observed that the direction of factor A indicated an inverse relation with the response R_2_. The other factors, B and C, were less prominent than factor A; however, they evidenced a synergistic effect on R_2_.

### 3.3. Selection of Optimized Formulation

**Figure 5:**
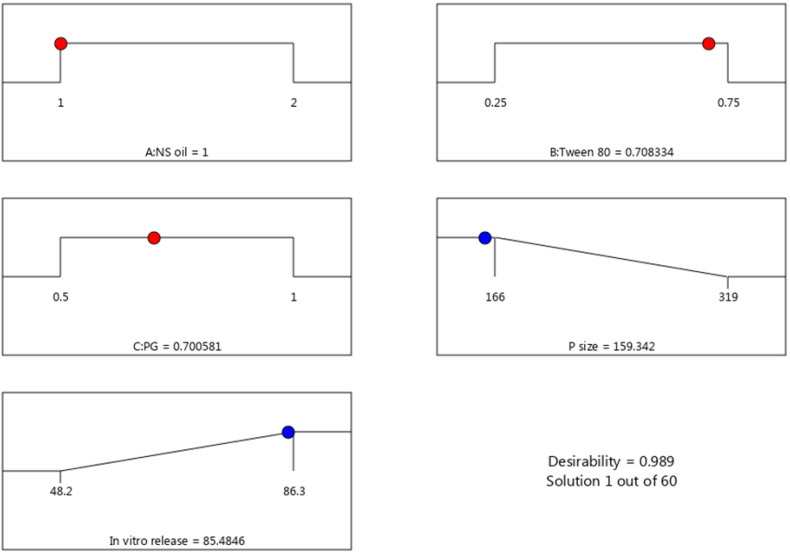
Optimization ramps for the independent variables (**A**–**C**) with the predicted values of responses (particle size and *in vitro* release) showing the desirability value.

Based on analysis of the response data, a numerical optimization and desirability approach were applied to determine the optimized NE formula. Numerical optimization was accomplished by directing the independent and the dependent variables toward certain required criteria that would achieve a higher desirability value [40]. The independent variables, A, B and C, were kept in a certain range, while R_1_ and R_2_ were adjusted to provide minimum and maximum values, as presented in Table 4. Based on the higher desirability value recorded of 0.989, the proposed values of the factors were 1 g NSO, 0.7 g Tween 80 and 0.7 g PG, as shown in Figure 5. The BBD predicted that these values would form an NE with a particle size of 159.49 nm and exhibiting an *in vitro* release of 85.48 %. Taking these predicted results into consideration, a new NE formula was prepared and evaluated for its response. It is notable that both values, predicted and observed, were in a close correspondence, as displayed in Table 4.

### 3.4. Characterization of the Optimized PGZ-Loaded NE

**Figure 6:**
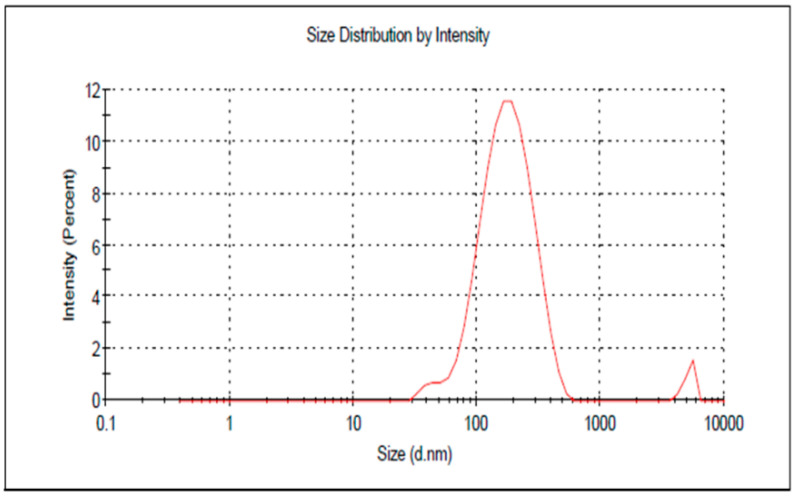
Particle size of optimized PGZ-loaded NE formulation.

**Figure 7:**
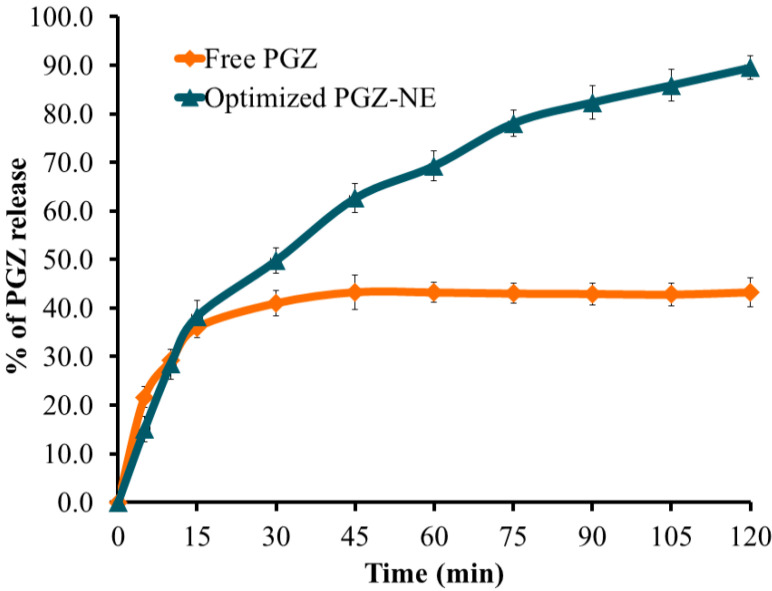
Profile illustrating *in vitro* release of optimized PGZ-loaded NE formulation.

The optimized formula was developed and evaluated for a particle size of 167.1 ± 3.43 nm with PDI of 0.308, as shown in Figure 6. The data indicated that the NE was homogenous and that the particles were distributed in a narrow range of sizes, which is a good indicator of formulation stability [41]. The *in vitro* release of PGZ from the optimized formula was compared to the release of PGZ from the free drug. As shown in Figure 7, it was notable that the PGZ dissolution was very poor. It demonstrated maximum release 45 min after the start of the experiment with a release value of 43.27 ± 3.6%. This could be attributed to the crystalline nature of the drug in addition to its poor solubility in the release media. The optimized PGZ-loaded NE showed an increase in the rate of dissolution of 89.5 ± 2.4% after 120 min. This was ascribed to the small particle size of the formulation that would provide a larger surface area contributing to enhanced release [42]. Additionally, it is well-known that the presence of a surfactant and co-surfactant would enhance the dissolution of the drug in the release media [43]. These factors imply that PGZ release would be significantly enhanced within the NE formulation.

### 3.5. Kinetic Study

Application of different kinetic models was used to determine the mechanism by which the drug would diffuse from the formula during *in vitro* release. On constructing different models representing the kinetics of drug release, it was noted that the Korsmeyer and Peppas model provided the best mechanism for PGZ release from the NE. It showed a linear correlation and displayed the highest value for the correlation coefficient (R^2^), being very close to 1 (0.9789), as seen in Table 5. The permeability exponent (n) for the PGZ-loaded NE formulation was found to be 1.0292, which was higher than 0.89. This indicates that the release from the NE system followed non-Fickian supercase II transport diffusion [44,45]. The result was in accordance with the findings of Rodríguez-Burneo et al., who found that the kinetic release of the drug from the prepared nanoemulsion fitted the Korsmeyer and Peppas model [46]. Additionally, Azhar et al., investigated the kinetic release of Kojic acid ester from the developed nanoemulsion and found it best fitted the Korsmeyer and Peppas model [44].

### 3.6. Drug Content

The drug content in optimized PGZ-loaded NE was measured and found to be 97.4 ± 2.17%. The result was in accordance with USP Pharmacopeial acceptance criteria (up to ± 15% of the label strength).

### 3.7. Scanning Electron Microscopy

**Figure 8:**
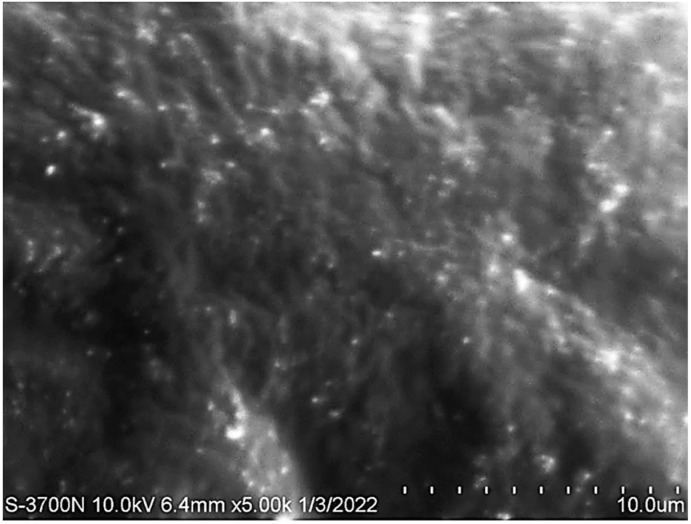
Scanning electron microscopy of the optimized PGZ-loaded NE formulation.

The morphology and the particle size of the optimized PGZ-loaded NE were evaluated using SEM. As is clear from Figure 8, the droplets of the NE appeared to be spherical in shape and were very close to the detected particle size, indicating a consistency between the results of both the SEM and the Malvern Zetasizer Nano ZS90.

### 3.8. Stability Study

**Figure 9:**
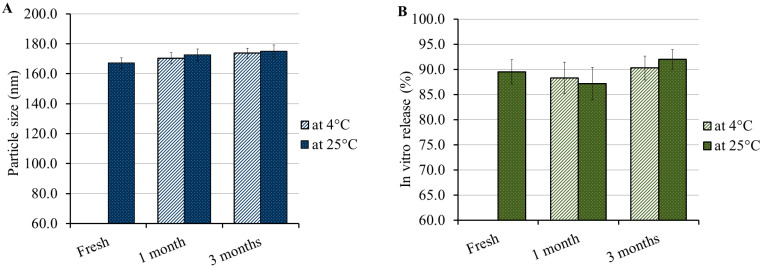
Stability study of optimized PGZ-loaded NE following storage for 1 and 3 months at two different temperatures 4 ± 3 °C and 25 ± 2 °C relative to (**A**) particle size and (**B**) *in vitro* release.

The formulation stability was checked by carrying out a stability test where the optimized PGZ-loaded NE formulation was stored in a well-closed container and kept at two different temperatures, 4 °C and 25 °C, for a period of 1 and 3 months. As shown in Figure 9, the results revealed that the formulation showed no variation in the examined parameters, particle size and *in vitro* release over the whole period of storage. The basis of the observed stability might be the presence of PEG-DSPE in the formulation which acted as a stabilizer. Previous investigations have found that conjugating formulations with PEG-DSPE prevents their aggregation and provides greater stability [47,48].

### 3.9. In Vivo Hypoglycemic Activity

**Figure 10:**
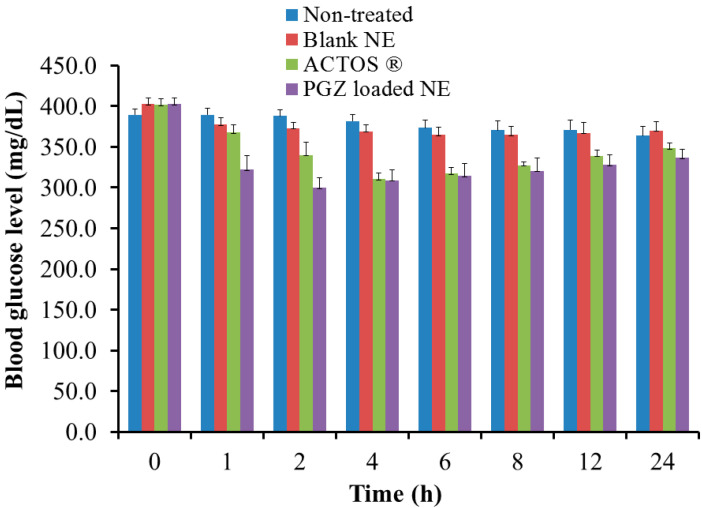
Profile representing the blood glucose level of rats induced with diabetes and treated with PGZ-loaded NE and blank NE compared to ACTOS^®^ formulation and non-treated rats.

Determination of the pharmacological hypoglycemic activity of the investigated formulations was conducted by measuring the blood glucose level in diabetic rats following oral administration of the formulations. As shown in Figure 10, blank NE showed significant reduction in blood glucose levels compared to the non-treated group at 2 and 4 h (*p* < 0.05). The ACTOS^®^-treated group showed a maximum percentage blood glucose level reduction of 77.18%, 4 h following drug administration. Interestingly, at the beginning of PGZ-loaded NE administration (1 and 2 h), the drug produced faster blood glucose level reduction than the drug in tablet formulation (ACTOS^®^), which could be due to better dissolution rates of PGZ from the NE form compared to the tablet form [49]. A maximum reduction in blood glucose level of 79.99% after 1 h of treatment was observed, followed by continuous hypoglycemic effects of the drug in NE form for 24 h. The PGZ-loaded-NE-treated group showed a rapid and significant reduction in PGZ at 1 and 2 h following drug administration compared to all other formulations under investigation (*p* < 0.05). The enhancement of the PGZ-loaded NE effect could be attributed to the synergistic effect of both drug and NSO during the first two hours of administration. This was consistent with the findings of Rahman et al., who found that *Nigella sativa* oil potentiated the hypoglycemic influence of Pioglitazone when given to rats induced with diabetes [50]. In conclusion, the hypoglycemic in vivo results of PGZ with NSO, showing a similar or slightly enhanced effect compared to ACTOS^®^, indicates that this formulation can be recommended as an alternative dosage form for special hypoglycemic patients.

## 4. Conclusions

The current study involved the successful development of *Nigella-sativa*-oil-loaded nanoemulsion formulations incorporating Pioglitazone for the treatment of high blood glucose levels. The formulations were investigated by applying a quality-by-design approach in which the influence of oil, surfactant and co-surfactant concentrations was examined for their effect on particle size and in vitro release. Based on the observed higher desirability, the optimized formulation was selected for further investigation. The optimized PGZ-loaded nanoemulsion formulation possessed appropriate characterizations and good stability on storage for a period of three months. Moreover, it showed a significant reduction in blood glucose levels, confirming the influence of *Nigella sativa* in potentiating the hypoglycemic effect of Pioglitazone and the potential value of nanoemulsions as a drug nanocarrier.

## Figures and Tables

**Table 1:** Factorial design independent variables with their level of variation and the examined dependent variables.

Independent Variable	Symbol	Level of Variation		
		Lowest (−1)	Central (0)	Highest (1)
NSO concentration (g)	A	1.0	1.5	2.0
Tween 80 concentration (g)	B	0.25	0.5	0.75
PG concentration (g)	C	0.5	0.75	1.0
Dependent variable	Symbol	Constraints		
Particle size (nm)	R_1_	Minimize		
*In Vitro* release (%)	R_2_	Maximize		

**Table 2:** Experimental runs and the observed responses of PGZ-loaded NE formulations using BBD.

Run	Selected Factors			Observed Responses	
	NSO Concentration (g)	Tween 80 Concentration (g)	PG Concentration (g)	P. Size (nm)	*In Vitro* (%)
F1	1	0.25	0.75	181 ± 4.6	78.6 ± 3.8
F2	1.5	0.5	0.75	188 ± 3.0	74.1 ± 2.8
F3	2	0.5	1	288 ± 4.6	54.0 ± 3.2
F4	1.5	0.5	0.75	195 ± 5.0	70.6 ± 2.2
F5	1.5	0.75	0.5	211 ± 4.6	71.9 ± 3.1
F6	1	0.75	0.75	166 ± 3.6	86.3 ± 2.9
F7	2	0.25	0.75	319 ± 5.4	48.2 ± 3.1
F8	1	0.5	1	170 ± 4.5	82.1 ± 3.4
F9	1.5	0.75	1	203 ± 4.4	68.1 ± 3.6
F10	1.5	0.5	0.75	190 ± 4.0	72.1 ± 2.9
F11	1	0.5	0.5	176 ± 4.6	80.3 ± 3.3
F12	2	0.5	0.5	300 ± 5.3	51.5 ± 2.4
F13	1.5	0.25	0.5	261 ± 4.5	63.5 ± 3.1
F14	2	0.75	0.75	270 ± 4.7	58.3 ± 2.6
F15	1.5	0.25	1	246 ± 4.9	66.5 ± 2.7

**Table 3:** Statistical analysis and fit statistics of all dependent variables R_1_ and R_2_.

Source	R_1_		R_2_	
	F-Value	*p*-Value	F-Value	*p*-Value
Model	153.39	<0.0001	58.36	0.0002
A	1090.58	<0.0001 *	476.08	<0.0001 *
B	114.75	0.0001 *	28.43	0.0031 *
C	7.83	0.0381 *	0.4349	0.5387
AB	10.76	0.0219 *	0.4090	0.5506
AC	0.3352	0.5877	0.1022	0.7621
BC	0.4562	0.5294	2.82	0.1540
A²	73.54	0.0004 *	6.40	0.0525
B²	54.32	0.0007 *	3.97	0.1029
C^2^	51.62	0.0008 *	9.10	0.0295 *
Lack of Fit	2.76	0.2759	1.24	0.4763
R²	0.9964		0.9906	
Adjusted R²	0.9899		0.9736	
Predicted R²	0.9519		0.8945	
Adequate Precision	37.8766		23.5140	
Model	Quadratic		Quadratic	
Remark	Suggested		Suggested	

A, NSO concentration (g); B, Tween 80 concentration (g); C, PG concentration (g); R_1_, particle size (nm); and R_2_, *in vitro* release (%); *, significant, *p* < 0.05.

**Table 4:** Predicted and observed values for the optimized PGZ-loaded NE formulation.

Dependent Variable	Symbol	Constraint
NSO concentration	A	In range
Tween 80 concentration	B	In range
PG concentration	C	In range
Response	Predicted values	Observed values
R_1_ (nm)	159.49 ± 5.18	167.1 ± 3.43
R_2_ (%)	85.48 ± 1.87	89.5 ± 2.38

**Table 5:** The correlation coefficient value for different kinetic modeling mechanisms.

Formula	Correlation Coefficient (R^2^)				Exponent (n) Value for Korsmeyer and Peppas
	Zero Order	First Order	Higuchi	Korsmeyer and Peppas	
Free PGZ	0.4729	0.5815	0.7619	0.9866	1.1188
PGZ-loaded NE	0.8853	0.7899	0.9674	0.9789	1.0292

## Data Availability

Not applicable.
